# Blood urea nitrogen to creatinine ratio is associated with in-hospital mortality in critically ill patients with venous thromboembolism: a retrospective cohort study

**DOI:** 10.3389/fcvm.2024.1400915

**Published:** 2024-06-13

**Authors:** Anju Puri, Mohan Giri, Huanhuan Huang, Qinghua Zhao

**Affiliations:** ^1^ Department of Nursing, The First Affiliated Hospital of Chongqing Medical University, Chongqing, China; ^2^ Department of Respiratory and Critical Care Medicine, The First Affiliated Hospital of Chongqing Medical University, Chongqing, China

**Keywords:** venous thromboembolism, blood urea nitrogen to creatinine ratio, intensive care unit, in-hospital mortality, critically ill

## Abstract

**Background:**

The relationship between the blood urea nitrogen to creatinine ratio (BCR) and the risk of in-hospital mortality among intensive care unit (ICU) patients diagnosed with venous thromboembolism (VTE) remains unclear. This study aimed to assess the relationship between BCR upon admission to the ICU and in-hospital mortality in critically ill patients with VTE.

**Methods:**

This retrospective cohort study included patients diagnosed with VTE from the Medical Information Mart for Intensive Care IV (MIMIC-IV) database. The primary endpoint was in-hospital mortality. Univariate and multivariate logistic regression analyses were conducted to evaluate the prognostic significance of the BCR. Receiver operating characteristic (ROC) curve analysis was utilized to determine the optimal cut-off value of BCR. Additionally, survival analysis using a Kaplan–Meier curve was performed.

**Results:**

A total of 2,560 patients were included, with a median age of 64.5 years, and 55.5% were male. Overall, the in-hospital mortality rate was 14.6%. The optimal cut-off value of the BCR for predicting in-hospital mortality in critically ill VTE patients was 26.84. The rate of in-hospital mortality among patients categorized in the high BCR group was significantly higher compared to those in the low BCR group (22.6% vs. 12.2%, *P* < 0.001). The multivariable logistic regression analysis results indicated that, even after accounting for potential confounding factors, patients with elevated BCR demonstrated a notably increased in-hospital mortality rate compared to those with lower BCR levels (all *P* < 0.05), regardless of the model used. Patients in the high BCR group exhibited a 77.77% higher risk of in-hospital mortality than those in the low BCR group [hazard ratio (HR): 1.7777; 95% CI: 1.4016–2.2547].

**Conclusion:**

An elevated BCR level was independently linked with an increased risk of in-hospital mortality among critically ill patients diagnosed with VTE. Given its widespread availability and ease of measurement, BCR could be a valuable tool for risk stratification and prognostic prediction in VTE patients.

## 1 Introduction

Venous thromboembolism (VTE), which includes deep vein thrombosis (DVT) and pulmonary embolism (PE), is a potentially preventable complication in critically ill patients (1). It is often associated with morbidity, mortality, and financial burdens, making it one of the significant contributors to the global burden of disease (1–3). The risk of VTE is significantly higher in critically ill patients compared to those hospitalized for other medical conditions (2). Although therapeutic advancements for VTE have resulted in improved patient outcomes in recent years, mortality rates continue to be higher, especially among critically ill patients (4–7). Prior research has identified several prognostic factors linked to in-hospital mortality in VTE patients, including age, preexisting comorbidities, VTE type, severity of illness, time to diagnosis and treatment, anticoagulation type, bleeding complications, and various laboratory parameters (8–10). Given the risk of VTE, identifying non-invasive and inexpensive tests for prompt recognition of high-risk patients with an increased mortality risk is key for improving patient care and reducing the impact of this potentially fatal condition.

Blood urea nitrogen (BUN) and creatinine are nitrogenous terminal products that indicate human renal function. Nevertheless, growing evidence suggests a possible correlation between these indicators and neurohormonal activity (11, 12). Dysregulated cardiorenal function and increased neurohormonal activation may contribute to higher levels of BUN and creatinine, which have been associated with increased mortality in several diseases (12–14). Many factors, such as medications, protein intake, muscle mass, and dehydration, influence BUN and creatinine levels (15, 16). Therefore, the BUN to creatinine ratio (BCR) is more valuable than either BUN or creatinine alone, as it is less prone to fluctuations and better reflects kidney function. The BCR is a useful predictor of outcomes in various diseases, and a high BCR is linked to increasing in-hospital mortality in critically ill patients, including those with septic shock (17), acute myocardial infarction (18), cerebral infarction (19), acute respiratory distress syndrome (20), cardiogenic shock (21), and COVID-19 (22). In patients with VTE, cardiorenal function and neurohumoral regulation are impaired due to various factors such as systemic hypoxia, activation of chemoreflex, hypercapnia, and inflammatory state, leading to elevated BUN and creatinine levels (23–27).

Despite the evidence mentioned above, to the best of our knowledge, no prior research has investigated the potential association between BCR and mortality rates in critically ill patients diagnosed with VTE. Therefore, utilizing the Medical Information Mart for Intensive Care-IV (MIMIC-IV) database (28), we hypothesized that an elevated BUN/creatinine ratio could increase the risk of in-hospital mortality among ICU patients with VTE.

## 2 Material and methods

### 2.1 Data source

All data used in this retrospective cohort study were extracted from the Medical Information Mart for Intensive Care-IV (MIMIC-IV) version 1.0, which includes electronic health records of adult patients admitted to the intensive care unit at Beth Israel Deaconess Medical Center in Boston, Massachusetts, between 2008 and 2019. Access to the database is granted to individuals who have completed the “Protecting Human Research Participants” training. For this study, author AP obtained the necessary certification and extracted the relevant data from the database (certification number: 61239194). Our research complied with the ethical principles outlined in the Helsinki Declaration. This study was approved by the Institutional Review Boards of Beth Israel Deaconess Medical Center and the Massachusetts Institute of Technology (Cambridge, MA, USA). Informed consent was not required as patient health information in this database was anonymized (28).

### 2.2 Study population

The diagnosis of VTE was based on the International Classification of Diseases, Ninth Revision (ICD-9) code, and the Tenth Revision (ICD-10) code. The ICD codes used for identifying patients with VTE are presented in Supplementary Table S1. Patients aged 18 years or older diagnosed with VTE were included in this study. Patients with multiple ICU admissions, those under 18 years old, individuals with an ICU stay of less than 24 h, and patients with missing data on BUN or creatinine were excluded from this study.

### 2.3 Data extraction

The Structured Query Language (SQL) was utilized to extract data using script codes obtained from the GitHub repository (https://github.com/MIT-LCP/mimic-iv). The following variables were collected: age, gender, length of stay (LOS) at the hospital, length of stay at the ICU, hospital death sign, Charlson comorbidity index (CCI), Simplified Acute Physiology Score II (SAPS II), laboratory tests on the day one of admission including hemoglobin, white blood cells (WBC), red blood cells (RBC), platelets, glucose, mean corpuscular hemoglobin concentration (MCHC), red blood cell distribution width (RDW), hematocrit, blood urea nitrogen (BUN), creatinine, bicarbonate, international normalized ratio (INR), prothrombin time (PT), and partial prothrombin time (PTT). The extracted preexisting comorbidities included hypertension, diabetes, congestive heart failure, coronary artery disease, renal disease, severe liver disease, obesity, malignant cancer, cerebrovascular disease, and chronic obstructive pulmonary disease (COPD). The extracted data also encompasses vital signs, such as heart rate, respiratory rate, mean arterial pressure (MAP), and peripheral oxygen saturation (SpO2). Information on whether patients required mechanical ventilation, received renal replacement therapy (RRT), or used diuretics was also extracted. The average value was used if a variable was assessed multiple times on the first day of admission. To mitigate potential bias, variables with missing values >20% were excluded. For variables with less than 20% missing values, the random forest imputation method from the missForest package in R software was used for imputation (29).

### 2.4 Outcomes

The primary outcome in the present study was in-hospital mortality. Secondary outcomes included length of ICU stay and length of hospital stay.

### 2.5 Statistical analysis

The normality of the variables was assessed using the Kolmogorov-Smirnov test. Results were presented as mean ± standard deviation (SD) for normally distributed data, and the independent sample *t*-test was employed for comparison between the groups. Conversely, variables were described as median with interquartile range (IQR) for non-normally distributed data, and the Mann–Whitney test was used for comparisons. Categorical variables were presented as total numbers and percentages, and the chi-square test or Fisher's exact test was utilized for analyses.

The characteristics of the patients included in the study were compared between the survival group and the deceased group. The optimal threshold values for BCR associated with in-hospital mortality were determined using the maximum Youden index through receiver operating characteristic (ROC) curve analysis. Subsequently, all patients with VTE were divided into two groups based on these cut-off values: the high BCR group and the low BCR group. Binomial logistic regression analysis was conducted to evaluate the influence of BCR on in-hospital mortality among patients with VTE. Variables with a *p*-value <0.1 in the univariate analysis and potential confounders identified through clinical expertise were included in the multivariate analysis. The crude model did not incorporate any adjustments to variables. In the multivariable analysis, three models (Model I, Model II, Model III) were developed to examine the association between BCR and in-hospital mortality. Survival curves were constructed utilizing the Kaplan-Meier method, with the log-rank test employed to compare survival rates between the high BCR and low BCR groups. All statistical analyses were conducted using SPSS version 26.0 and MedCalc version 19.6 software. A *p*-value of less than 0.05 was considered statistically significant.

## 3 Results

### 3.1 Baseline characteristics of the participants

**Figure 1:**
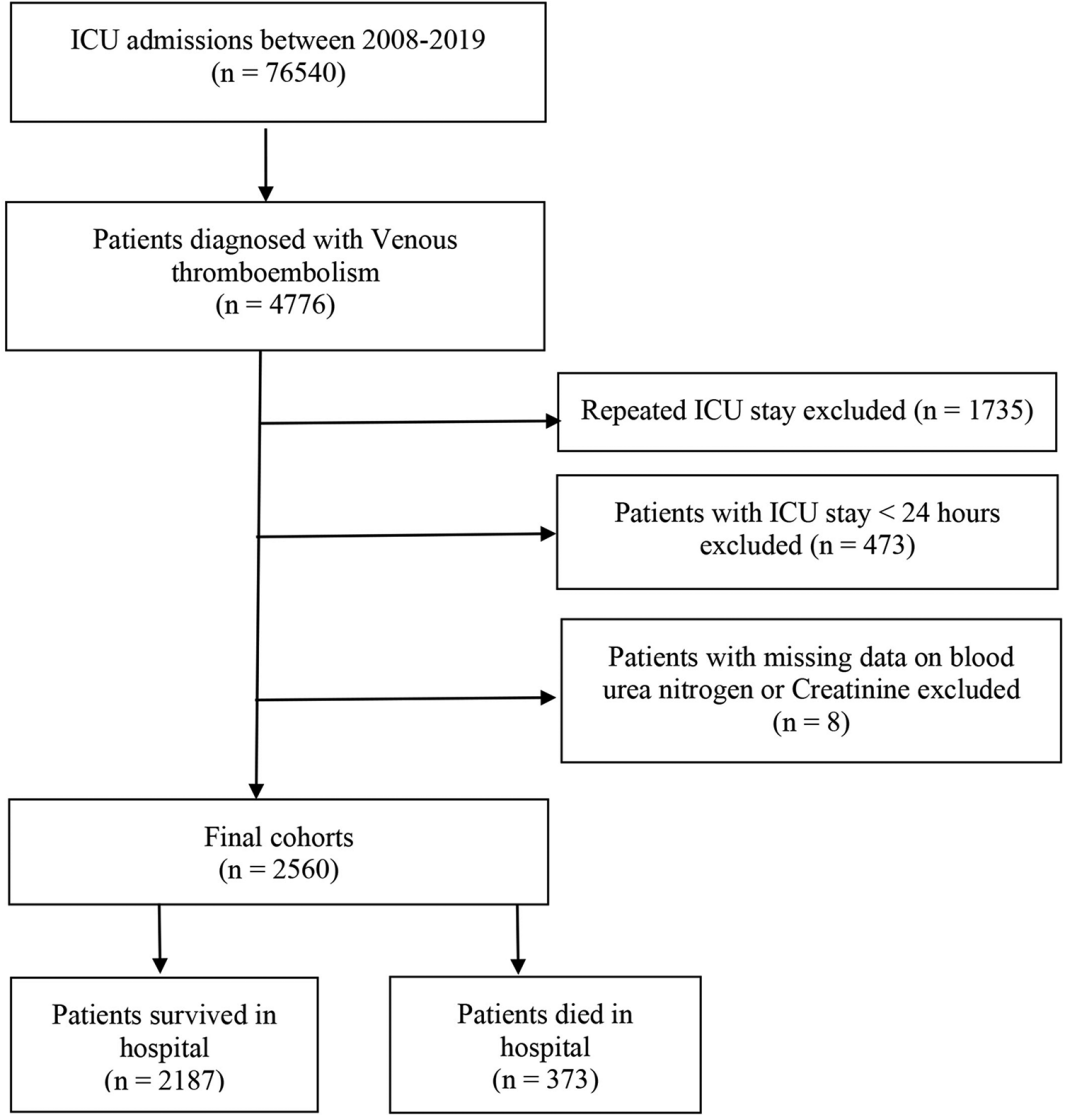
Flowchart for selecting analyzed participants.

This study included a total of 2,560 critically ill patients diagnosed with VTE. The flowchart for patient screening is depicted in Figure 1. The median age of the participants with VTE was 64.5 years, and 55.5% were male. The in-hospital mortality rate was 14.6% (373/2,560). Table 1 summarizes the baseline characteristics of both the survival and deceased groups. Compared with patients in the survival group, the patients in the death group tended to be older. They had a higher proportion of diabetes, congestive heart failure, renal disease, severe liver disease, obesity, and cancer (all *P* < 0.05). Individuals in the deceased group exhibited elevated levels of creatinine, white blood cells (WBC), red cell distribution width (RDW), heart rate, and respiratory rate compared to those in the survival group (all *p* < 0.05). Conversely, they demonstrated lower mean arterial pressure, hemoglobin, red blood cell (RBC) count, platelet count, mean corpuscular hemoglobin concentration (MCHC), hematocrit, and bicarbonate levels (all *p* < 0.05). The CCI and SAPS II scores were higher in the deceased group, whereas the length of hospital stay was shorter compared to the survival group (all *p* < 0.05). Moreover, patients in the death group were more likely to receive RRT and diuretics, and they more frequently required mechanical ventilation during their hospital stay compared to the survival group (all *p* < 0.05). We found that patients in the death group had significantly higher levels of BUN, creatinine, and BCR when compared with the survival group (Table 1).

**Table 1:** Demographic and clinical characteristics of patients with venous thromboembolism.

Characteristics	Total (*n* = 2,560)	Survival (*n* = 2,187)	Death (*n* = 373)	*p*-value
Age, year	64.5 (53–75.5)	64 (52–74.5)	69 (60–79)	<0.001
Gender, male	1,420 (55.5%)	1,226 (56.1)	194 (52)	0.146
CCI	6 (4–8)	5 (3–7)	7 (6–10)	<0.001
SAPS II Score	36 (27–46)	34 (25–43)	49 (38–61)	<0.001
Laboratory tests				<0.001
Hemoglobin, g/dl	10.7 (9.2–12.4)	10.8 (9.4–12.5)	10 (8.6–11.6)	<0.001
WBC, 109/L	11.2 (8.3–15.1)	11.1 (8.2–14.6)	12.7 (8.7–18.4)	<0.001
RBC, 109/L	3.6 (3.1–4.2)	3.6 (3.2–4.2)	3.4 (2.9–3.9)	<0.001
Platelets, 109/L	192 (133–264)	194.5 (137–264.5)	170 (100.3–261.7)	<0.001
Glucose, mg/dl	129 (109–160.5)	127.5 (108.5–159)	137.5 (111.5–172.5)	<0.001
MCHC, g/dl	33.1 (32.1–34.1)	33.1 (32.2–34.2)	32.7 (31.6–33.7)	<0.001
RDW, %	14.8 (13.7–16.5)	14.6 (13.6–16.2)	16 (14.6–17.8)	<0.001
Hematocrit, %	32.2 (28–37)	32.5 (28.3–37.2)	30.8 (26.6–35.1)	<0.001
BUN, mg/dl	19 (13–30)	18 (12.5–28)	28 (18–43)	<0.001
Creatinine, ng/dl	1 (0.7–1.4)	0.9 (0.7–1.3)	1.2 (0.8–2)	0.040
BUN/Cr ratio	19.1 (14.3–26.1)	18.7 (14.1–25.1)	22 (15.5–30.8)	<0.001
Bicarbonate, mEq/L	23 (20–25.5)	23 (20.5–25.5)	21.5 (18–24)	<0.001
Coagulation tests				<0.001
INR	1.3 (1.2–1.5)	1.3 (1.2–1.5)	1.4 (1.2–1.9)	<0.001
PT	14.4 (12.9–16.8)	14.3 (12.8–16.4)	15.8 (13.6–20.3)	<0.001
PTT	34.4 (28.8–59)	35.6 (28.5–57.7)	41.1 (30.2–67.4)	<0.001
Comorbidities				
Hypertension	1,014 (39.6)	881 (40.3)	133 (35.7)	0.091
Diabetes	623 (24.3)	514 (23.5)	109 (29.2)	0.017
Congestive heart failure	593 (23.2)	482 (22)	111 (29.8)	0.001
Coronary artery disease	464 (18.1)	383 (17.5)	81 (21.7)	0.051
Renal disease	385 (15)	304 (13.9)	81 (21.7)	<0.001
Severe liver disease	246 (9.6)	184 (8.4)	62 (16.6)	<0.001
Obesity	334 (13)	299 (13.7)	35 (9.4)	0.023
Malignant cancer	586 (22.9)	445 (20.3)	141 (37.8)	0.000
Cerebrovascular disease	392 (15.3)	325 (14.9)	67 (18)	0.124
COPD	649 (25.4)	544 (24.9)	105 (28.2)	0.179
Monitoring parameters				
Heart rate, Bpm	89 (78–102)	89 (77–101)	93 (81–104)	<0.001
MAP, mmHg	78 (71–86)	78 (72–87)	75 (68–82)	<0.001
RR, breaths/minutes	19 (17–23)	19 (17–22)	21 (18–24)	<0.001
SpO2,%	97 (95–98)	97 (96–98)	97 (95–98)	0.005
Intervention				
Mechanical ventilation	991 (38.5)	804 (36.8)	187 (50.1)	<0.001
Diuretic use	382 (14.9)	305 (13.9)	77 (20.6)	0.001
RRT	205 (8)	131 (6)	74 (19.8)	<0.001
Outcomes				
LOS hospital, day	12.2 (6.9–20.9)	12.4 (7–20.9)	11.8 (5.3–20.5)	0.018
LOS ICU, day	3.2 (1.9–6.9)	3 (1.9–6.3)	4.7 (2.4–10.1)	0.000

Values are expressed as the median (Interquartile range) or *n* (%). CCI, Charlson Comorbidity Index; SAPS, the simplified acute physiology score; WBC, white blood cells; RBC, red blood cell; MCHC, mean corpuscular hemoglobin concentration; RDW, red blood cell distribution width; BUN, blood urea nitrogen; INR, International normalized ratio; PT, prothrombin time; PTT, partial thromboplastin time; RRT, renal replacement therapy; MAP, mean arterial pressure; RR, respiratory rate; LOS, length of stay; ICU, intensive care unit.

### 3.2 The prognostic significance of BCR

**Figure 2:**
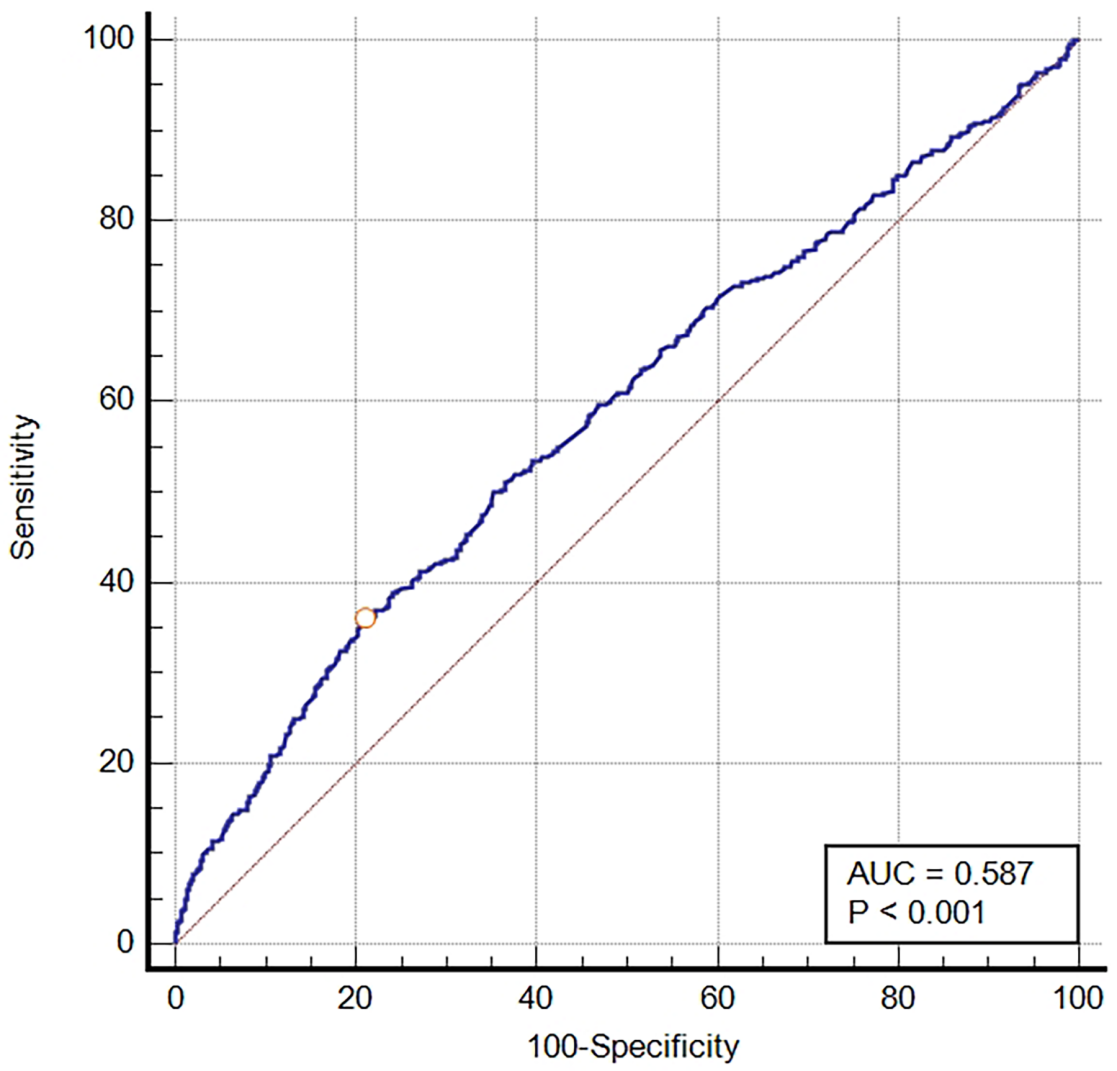
The ROC curve of predictive performance of BCR for in-hospital mortality. AUC, area under the curve; BCR, blood urea nitrogen to creatinine ratio; ROC, receiver operator characteristic curve.

The ROC curve was generated for BCR to predict in-hospital mortality in critically ill patients with VTE, and the area under the ROC curve was 0.587 (95% CI: 0.568–0.607, *P* < 0.001) (Figure 2). The optimal cut-off value of BCR to predict survival status was 26.84, with a sensitivity of 35.93% and a specificity of 79.06%. Using this cut-off value, patients were categorized into two groups: the low BCR group (≤26.84, *n* = 1,966) and the high BCR group (>26.84, *n* = 594). Table 2 displays the baseline characteristics of the low BCR and high BCR groups. In comparison with patients in the low BCR group, those in the high BCR group exhibited a significantly higher in-hospital mortality rate (22.6% vs. 12.2%, *P* < 0.001). Patients with a higher BCR (>26.84) were more likely to have comorbid diseases, including congestive heart failure, coronary artery disease, severe liver disease, cancer, and COPD, as well as higher CCI, SAPS II scores, hemoglobin levels, platelet counts, RBC counts, MCHC, RDW, hematocrit, PT, and INR levels compared to those with a low BCR (≤26.84) (all *P* < 0.05). Additionally, individuals with a BCR >26.84 were prone to experiencing prolonged hospital and ICU stays (*p* < 0.05). They also exhibited a higher likelihood of receiving diuretic therapy, mechanical ventilation, and renal replacement therapy compared to those with a low BCR ≤26.84 (all *P* < 0.05) (Table 2).

**Table 2:** Baseline characteristics of BUN/creatinine ratio groups in patients with venous thromboembolism.

Characteristics	Total (*n* = 2,560	Low BCR ≤ 26.84 (*n* = 1,966)	High BCR >26.84 (*n* = 594)	*P*
Age, year	64.5 (53–75.5)	63 (51–74)	69 (58–78)	<0.001
Gender, male	1,420 (55.5%)	1,440 (55.5)	221 (51.2)	0.095
CCI	6 (4–8)	5 (3–8)	6 (5–8)	<0.001
SAPS II Score	36 (27–46)	33 (25–44)	40 (33–50)	<0.001
Laboratory tests				
Hemoglobin, g/dl	10.7 (9.2–12.4)	10.8 (9.4–12.6)	10.1 (8.8–11.7)	<0.001
WBC, 109/L	11.2 (8.3–15.1)	11.1 (8.3–14.8)	11.6 (8.2–16.3)	0.106
RBC, 109/L	3.6 (3.1–4.2)	3.7 (3.2–4.2)	3.4 (3.0–3.9)	<0.001
Platelets, 109/l	192 (133–264)	194.4 (139.0–263.3)	182.3 (114.2–265.8)	0.001
Glucose, mg/dl	129 (109–160.5)	129.0 (109.0–160.0)	129.0 (110.0–165.0)	0.331
MCHC, g/dl	33.1 (32.1–34.1)	33.2 (32.2–34.2)	32.9 (31.8–33.9)	<0.001
RDW, %	14.8 (13.7–16.5)	14.6 (13.5–16.2)	15.6 (14.3–17.6)	<0.001
Hematocrit, %	32.2 (28–37)	32.7 (28.3–37.4)	30.6 (27.1–35.7)	<0.001
Bicarbonate, mEq/L	23 (20–25.5)	23.0 (20.0–25.0)	23.0 (20.0–26.0)	0.056
Coagulation tests				
INR	1.3 (1.2–1.5)	1.3 (1.2–1.5)	1.4 (1.2–1.7)	<0.001
PT	14.4 (12.9–16.8)	14.3 (12.8–16.5)	14.9 (13.2–17.8)	<0.001
PTT	34.4 (28.8–59)	37 (28.9–60.2)	34.5 (28.2–55.9)	0.026
Comorbidities				
Hypertension	1,014 (39.6)	778 (39.6)	236 (39.7)	0.945
Diabetes	623 (24.3)	478 (24.3)	145 (24.4)	0.961
Congestive heart failure	593 (23.2)	422 (21.5)	171 (28.8)	0.000
Coronary artery disease	464 (18.1)	336 (17.1)	128 (21.5)	0.013
Renal disease	385 (15)	317 (16.1)	68 (11.4)	0.005
Severe liver disease	246 (9.6)	164 (8.3)	82 (13.8)	<0.001
Obesity	334 (13)	268 (13.6)	66 (11.1)	0.110
Malignant cancer	586 (22.9)	417 (21.2)	169 (28.5)	<0.001
Cerebrovascular disease	392 (15.3)	314 (16)	78 (13.1)	0.092
COPD	649 (25.4)	476 (24.2)	173 (29.1)	0.016
Monitoring parameters				
Heart rate, Bpm	89 (78–102)	89 (77–101)	91 (78–103)	0.084
MAP, mmHg	78 (71–86)	79 (72–87)	76 (69–82)	<0.001
RR, breaths/minutes	19 (17–23)	19 (17–22)	20 (17–23)	0.015
SpO2,%	97 (95–98)	97 (96–98)	97 (95–98)	0.159
Intervention				
Mechanical ventilation	991 (38.5)	757 (38.5)	234 (39.4)	0.697
Diuretic use	382 (14.9)	273 (13.9)	109 (18.4)	0.007
RRT	205 (8)	175 (8.9)	30 (5.1)	0.002
Outcomes				
Length of hospital stay, days	12.2 (6.9–20.9)	12.4 (7–20.9)	11.8 (5.3–20.5)	0.018
Length of ICU stay, days	3.2 (1.9–6.9)	3 (1.9–6.3)	4.7 (2.4–10.1)	<0.001
In-hospital mortality, *n* (%)	373 (14.6)	239 (12.2)	134 (22.6)	<0.001

Values are expressed as the median (Interquartile range) or *n* (%). CCI, Charlson Comorbidity Index; SAPS, the simplified acute physiology score; WBC, white blood cells; RBC, red blood cell; MCHC, mean corpuscular hemoglobin concentration; RDW, red blood cell distribution width; BUN, blood urea nitrogen; INR, international normalized ratio; PT, prothrombin time; PTT, partial thromboplastin time; RRT, renal replacement therapy; MAP, mean arterial pressure; RR, respiratory rate; LOS, length of stay; ICU, intensive care unit.

### 3.3 Association between BCR and in-hospital mortality in patients with VTE

The results of the univariable logistic regression analysis are presented in Table 3. Additionally, Table 4 illustrates the unadjusted and multivariable-adjusted correlations between BCR and in-hospital mortality. The logistic regression model was employed to evaluate the impact of exposure variables on the outcome measures while adjusting for covariates. The crude model was not adjusted. In Model I, age and gender were incorporated as covariates to account for potential confounders. We adjusted for 11 variables in Model II, including hypertension, diabetes, congestive heart failure, coronary artery disease, renal disease, severe liver disease, obesity, malignant cancer, mechanical ventilation, diuretic use, and renal replacement therapy. Model III was adjusted for 18 variables, including CCI, SAPS II Score, hemoglobin, WBC, RBC, platelets, glucose, MCHC, RDW, hematocrit, bicarbonate, INR, PT, APTT, HR, MAP, RR, and SpO2. A statistically significant positive association was observed between the BCR (a continuous variable) and in-hospital mortality across all models: Crude Model: OR = 1.029, 95% CI: 1.020–1.038, *P* < 0.001; Model I: OR = 1.024, 95% CI: 1.014–1.033, *P* < 0.001; Model II: OR = 1.032, 95% CI: 1.023–1.042, *P* < 0.001; Model III: OR = 1.016, 95% CI: 1.006–1.026, *P* = 0.002). Additionally, compared to the low BCR (≤26.84) group, in-hospital mortality was significantly higher in the high BCR (>26.84) group across different models: Crude Model (OR = 2.105, 95% CI: 1.664–2.663, *P* < 0.001); Model I (OR = 1.874, 95% CI: 1.874–2.381, *P* < 0.001); Model II (OR = 2.091, 95% CI: 1.626–2.688, *P* < 0.001); and Model III (OR = 1.420, 95% CI: 1.086–1.857, *P* = 0.010) (Table 4).

**Table 3:** Univariate logistic regression analyses for in–hospital mortality in patients with VTE.

Variable	OR (95% CI)	*P* value
Age, year	1.025 (1.018–1.033)	<0.001
Gender, male	0.850 (0.682–1.059)	0.146
CCI	1.229 (1.187–1.272)	<0.001
SAPS II Score	1.064 (1.056–1.072)	<0.001
Hemoglobin, g/dl	0.849 (0.804–0.897)	<0.001
WBC, 109/L	1.014 (1.005–1.022)	0.001
RBC, 109/L	0.629 (0.537–0.737)	<0.001
Platelets, 109/L	0.999 (0.998–1.000)	0.014
Glucose, mg/dl	1.004 (1.003–1.006)	<0.001
MCHC, g/dl	0.822 (0.768–0.880)	<0.001
RDW, %	1.195 (1.149–1.242)	<0.001
Hematocrit, %	0.958 (0.941–0.976)	<0.001
Bicarbonate, mEq/L	0.899 (0.875–0.924)	0.056
INR	1.495 (1.312–1.702)	<0.001
PT	1.041 (1.028–1.054)	<0.001
PTT	1.007 (1.003–1.011)	<0.001
Hypertension	0.822 (0.654–1.032)	0.092
Diabetes	1.344 (1.053–1.715)	0.018
Congestive heart failure	1.499 (1.174–1.913)	0.001
Coronary artery disease	1.307 (0.998–1.711)	0.052
Renal disease	1.718 (1.306–2.261)	<0.001
Severe liver disease	2.170 (1.589–2.964)	<0.001
Obesity	0.654 (0.452–0.945)	0.024
Malignant cancer	2.379 (1.883–3.006)	<0.001
Cerebrovascular disease	1.254 (0.939–1.676)	0.125
COPD	1.183 (0.926–1.513)	0.179
Heart rate, Bpm	1.012 (1.005–1.019)	<0.001
MAP, mmHg	0.959 (0.948–0.970)	<0.001
RR, breaths/minutes	1.060 (1.034–1.087)	<0.001
SpO2,%	0.914 (0.876–0.954)	<0.001
Mechanical ventilation	1.729 (1.387–2.157)	<0.001
Diuretic use	1.605 (1.215–2.120)	0.001
RRT	3.884 (2.850–5.295)	<0.001
Length of hospital stay, days	1.004 (0.998–1.009)	0.216
Length of ICU stay, days	1.029 (1.018–1.040)	<0.001

VTE; venous thromboembolism; OR, odds ration; CCI, Charlson Comorbidity Index; SAPS, the simplified acute physiology score; WBC, white blood cells; RBC, red blood cell; MCHC, mean corpuscular hemoglobin concentration; RDW, red blood cell distribution width; BUN, blood urea nitrogen; INR, international normalized ratio; PT, prothrombin time; PTT, partial thromboplastin time; RRT, renal replacement therapy; MAP, mean arterial pressure; RR, respiratory rate; LOS, length of stay; ICU, intensive care unit.

**Table 4:** Multivariable logistic regression analyses for in-hospital mortality in patients with venous thromboembolism.

Characteristic	Crude model		Model I		Model II		Model III	
	OR (95% CI)	*p*-value	OR (95% CI)	*p*-value	OR (95% CI)	*p*-value	OR (95% CI)	*p*-value
BCR	1.029 (1.020–1.038)	<0.001	1.024 (1.014–1.033)	<0.001	1.032 (1.023–1.042)	<0.001	1.016 (1.006–1.026)	0.002
Low BCR	1 (Reference)		1 (Reference)		1 (Reference)		1 (Reference)	
High BCR	2.105 (1.664–2.663)	<0.001	1.874 (1.475–2.381)	<0.001	2.091 (1.626–2.688)	<0.001	1.420 (1.086–1.857)	0.010

The crude model did not adjust any of the variables.

Model I made adjustments to the variables of age and gender.

Model II made adjustments to 11 variables, including hypertension, diabetes, congestive heart failure, coronary artery disease, renal disease, severe liver disease, obesity, malignant cancer, mechanical ventilation, diuretic use, and renal replacement therapy.

Model III made further adjustments to 18 variables, including CCI, SAPS II Score, hemoglobin, WBC, RBC, platelets, glucose, MCHC, RDW, hematocrit, bicarbonate, INR, PT, PTT, HR, MAP, RR, and SpO2.

### 3.4 Kaplan–Meier survival analysis of the cohort by BCR groups

**Figure 3:**
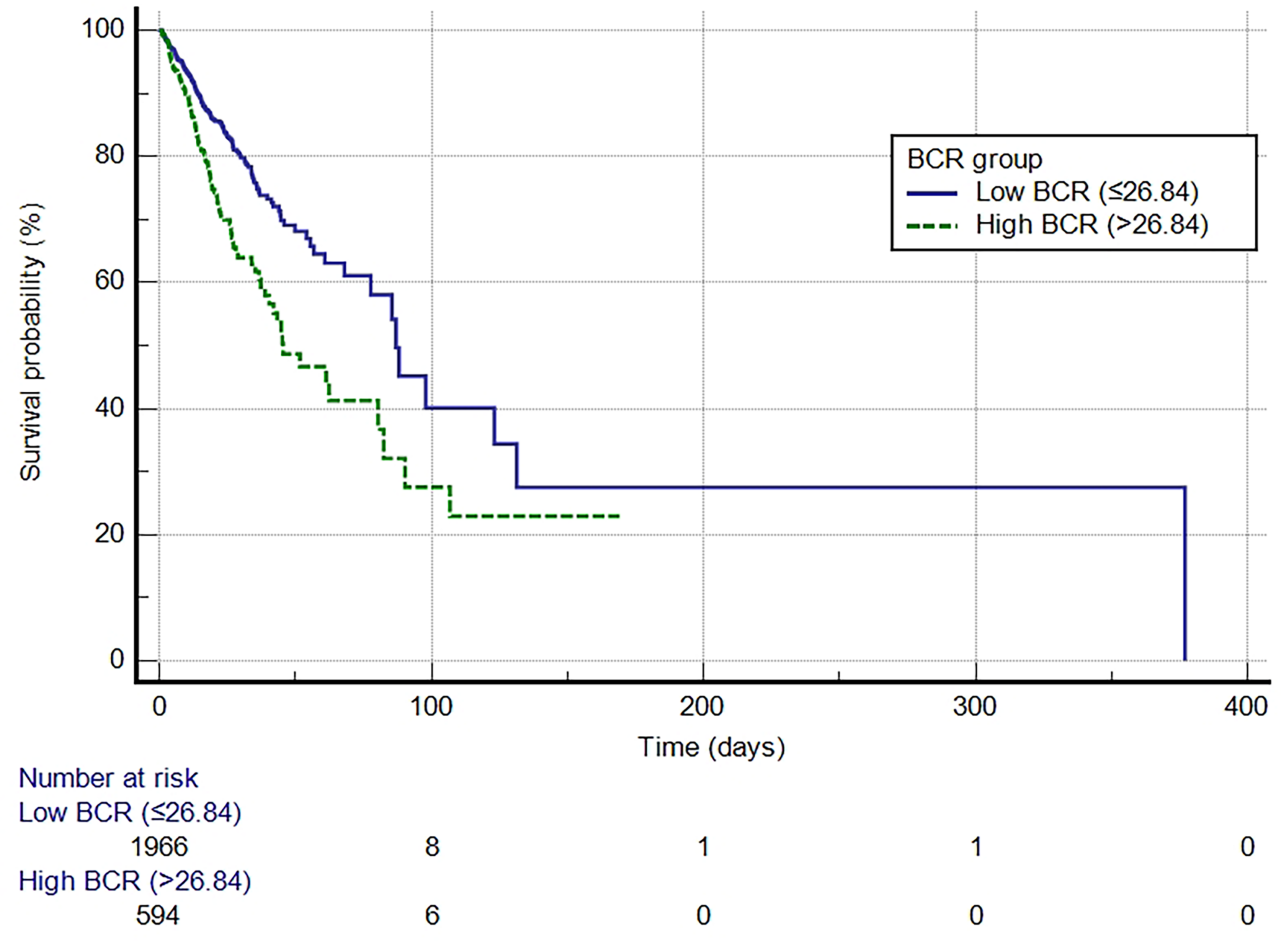
Kaplan-Meier survival curve for in-hospital mortality for the high and low BCR groups. BCR, blood urea nitrogen to creatinine ratio.

A Kaplan-Meier curve was generated to demonstrate the survival outcomes of patients with VTE in both the high BCR and low BCR groups (Figure 3). The median survival time for the low BCR group was 86.976 days (95% CI: 77.704–377.026), whereas for the high BCR group, it was 45.477 days (95% CI: 38.876–80.425). This difference was statistically significant (log-rank test, *P* < 0.0001). In comparison to the low BCR group, the hazard ratio (HR) for the high BCR group was 1.7777 (95% CI: 1.4016–2.2547).

### 3.5 Subgroup analysis

Table 5 shows the results of the subgroup analysis. High BCR was consistently associated with the risk of in-hospital mortality among most subgroups (all *P* for interaction >0.05), except for age (interaction *P* = 0.042), diabetes (interaction *P* = 0.004), and severe liver disease (interaction *P* = 0.002).

**Table 5:** Subgroup analysis of the association between BCR and in-hospital mortality in VTE patients.

Subgroup	*N*	OR (95% CI) High BCR >26.84	*P* for interaction
Gender	** **		0.282
Male	1,420	2.371 (1.703–3.301)	
Female	1,140	1.830 (1.309–2.559)	
Age	** **		0.042
<65	1,280	2.722 (1.853–3.998)	
≥65	1,280	1.639 (1.215–2.210)	
Hypertension	** **		0.359
Yes	1,014	2.432 (1.656–3.571)	
No	1,546	1.937 (1.438–2.609)	
Diabetes	** **		0.004
Yes	623	1.173 (0.728–1.889)	
No	1,937	2.601 (1.979–3.419)	
Congestive heart failure	** **		0.096
Yes	593	1.510 (0.976–2.335)	
No	1,967	2.340 (1.769–3.095)	
Coronary artery disease	** **		0.084
Yes	464	3.067 (1.868–5.038)	
No	2,096	1.863 (1.422–2.440)	
Renal disease	** **		0.251
Yes	385	1.595 (0.877–2.900)	
No	2,175	2.327 (1.796–3.015)	
Severe liver disease	** **		0.002
Yes	246	0.849 (0.457–1.577)	
No	2,314	2.374 (1.839–3.063)	
Obesity			0.363
Yes	334	1.470 (0.653–3.307)	
No	2,226	2.162 (1.690–2.768)	
Malignant cancer			0.650
Yes	586	1.857 (1.245–2.770)	
No	1,974	2.083 (1.550–2.801)	
Cerebrovascular disease			0.180
Yes	392	3.055 (1.717–5.434)	
No	1,862	1.978 (1.527–2.561)	
COPD			0.066
Yes	649	1.471 (0.939–2.306)	
No	1,911	2.403 (1.823–3.168)	

The reference group was Low BCR group; BCR, Blood urea nitrogen to creatinine ratio; VTE, venous thromboembolism.

### 3.6 Analysis based on types of VTE (DVT and PE)

All VTE patients were divided into groups based on their diagnosis of either DVT or PE. The results were then analyzed separately for each group. Table 6 summarizes the baseline demographic and clinical characteristics of patients with DVT and PE. For patients with DVT, the comparison between survival and deceased cohorts revealed significant differences. Deceased patients were significantly older with higher CCI and SAPS II scores (*p* < 0.001). Laboratory tests showed decreased hemoglobin, RBC, and platelet levels but increased WBC, glucose, RDW, BUN, creatinine, and BUN/Cr ratio in deceased individuals (all *p* < 0.05). Moreover, deceased individuals demonstrated lower bicarbonate levels and higher INR PT and APTT values (all *p* < 0.05). Comorbidities such as congestive heart failure, malignant cancer, severe liver disease, and renal disease were more prevalent among deceased DVT patients (*p* < 0.05). Deceased DVT patients more often received renal replacement therapy (*p* < 0.001). Furthermore, deceased DVT patients had longer ICU stays (*p* < 0.01). DVT patients in the high BCR group (BCR > 26.84) demonstrated a significantly higher mortality rate compared to those in the low BCR group (*p* < 0.001) (Table 6). In patients with DVT, the crude logistic regression analysis showed that those in the high BCR group have a significantly higher risk of in-hospital mortality compared to those in the low BCR group, with an OR of 2.170 (95% CI: 1.612–2.921, *P* < 0.001). Even after adjusting for potential confounders, the association remained significant, with an adjusted odds ratio (aOR) of 1.872 (95% CI: 1.330–2.634; *p* < 0.001) (Supplementary Table S2). In patients with DVT, the Kaplan-Meier survival analysis revealed that the median survival time for the high BCR group was 44.840 days (95% CI: 35.315–80.825), which is significantly shorter than the 86.976 days (95% CI: 68.013–377.026) for the low BCR group. The high BCR group had an 81% higher risk of in-hospital mortality (HR: 1.8145, 95% CI: 1.3432–2.4510). The log-rank test indicated a statistically significant difference in survival between the two groups (Logrank *p* < 0.0001) (Supplementary Figure S1).

**Table 6:** Demographic and clinical characteristics of patients with deep vein thrombosis and pulmonary embolism.

Characteristics	Deep vein thrombosis			Pulmonary embolism		
	Survival (*n* = 1,366)	Death (*n* = 231)	*P*	Survival (*n* = 821)	Death (*n* = 142)	*P value*
Age, year	63 (53–74)	70 (60–80)	<0.001	64 (51–75)	69 (60–78)	<0.001
Gender, male	822 (60.2%)	132 (57.1%)	0.385	404 (49.2)	62 (43.7)	0.222
CCI	5 (4–7)	8 (6–10)	<0.001	5 (3–7)	7 (5–10)	<0.001
SAPS II Score	35 (26–43)	48 (38–61)	<0.001	32 (24–42)	50 (38–61)	<0.001
Laboratory tests						
Hemoglobin, g/dl	10.7 (9.4–12.5)	10.1 (8.8–11.3)	<0.001	10.9 (9.3–12.5)	9.8 (8.5–11.9)	0.001
WBC, 10^9^/L	11 (7.9–14.6)	12.2 (8.2–18.3)	0.007	11.2 (8.6–14.5)	13.9 (9.5–18.9)	<0.001
RBC, 109/L	3.6 (3.1–4.1)	3.3 (2.9–3.8)	<0.001	3.7 (3.2–4.3)	3.4 (2.9–4.1)	<0.001
Platelets, 109/L	185.1 (129.5–252)	161 (93.3–248)	0.001	211.2 (152.5–279)	192.8 (110–273.7)	0.022
Glucose, mg/dl	128.3 (108.5–159.5)	137.5 (111.5–167.3)	0.041	126.5 (108.5–158)	137.8 (113–192)	<0.001
MCHC, g/dl	33.4 (32.4–34.4)	32.9 (31.8–34)	<0.001	32.7 (31.8–33.7)	32.3 (31.4–33.3)	0.001
RDW, %	14.6 (13.6–16.2)	16.5 (14.8–18.4)	<0.001	14.6 (13.5–16.2)	15.6 (14.4–17.4)	<0.001
Hematocrit, %	32.1 (28.1–36.9)	30.3 (26.5–34.6)	<0.001	33.2 (28.7–37.8)	31.1 (26.9–36.6)	0.007
BUN, mg/dl	18.5 (13–30)	30 (18.5–46.5)	<0.001	17 (12.5–25.5)	24 (15–37)	<0.001
Creatinine, ng/dl	1 (0.8–1.4)	1.3 (0.9–2.2)	<0.001	0.9 (0.7–1.2)	1.1 (0.8–1.7)	<0.001
BUN/Cr ratio	18.5 (14–25.4)	22.2 (14.9–31.9)	<0.001	19 (14.3–25)	21.1 (15.6–29.8)	0.002
Bicarbonate, mEq/L	23 (20.5–25.5)	21.5 (18–24)	<0.001	23 (20.5–25.5)	21.5 (18–24.5)	<0.001
Coagulation tests						
INR	1.3 (1.2–1.5)	1.5 (1.3–2)	<0.001	1.3 (1.2–1.5)	1.4 (1.2–1.7)	<0.001
PT	14.4 (12.8–16.6)	16.4 (14–21.7)	<0.001	14.1 (12.8–16)	15.1 (13.3–17.8)	<0.001
PTT	33.9 (28.2–49.2)	41.1 (31.6–59.1)	<0.001	42.4 (29.1–76.6)	41.8 (29.7–87.8)	0.395
Comorbidities						
Hypertension	563 (41.2%)	74 (32%)	0.008	318 (38.7)	59 (41.5)	0.526
Diabetes	341 (25.0)	68 (29.4)	0.150	173 (21.1)	41 (28.9)	0.039
Congestive heart failure	285 (20.9)	62 (26.8)	0.042	197 (24.0)	49 (34.5)	0.008
Coronary artery disease	278 (20.4)	48 (20.8)	0.881	105 (12.8)	33 (23.2)	0.001
Renal disease	207 (15.2)	55 (23.8)	0.001	97 (11.8)	26 (18.3)	0.032
Severe liver disease	168 (12.3)	57 (24.7)	<0.001	16 (1.9)	5 (3.5)	0.236
Obesity	150 (11)	16 (6.9)	0.062	149 (18.1)	19 (13.4)	0.167
Malignant cancer	264 (19.3)	90 (39)	<0.001	181 (22.0))	51 (35.9	<0.001
Cerebrovascular disease	222 (16.3)	35 (15.2)	0.674	103 (12.5)	32 (22.5)	0.002
COPD	318 (23.3)	60 (26.0)	0.373	226 (27.5)	45 (31.7)	0.308
Monitoring Parameters						
Heart rate, Bpm	87 (76–100)	92 (82–104)	<0.001	92 (79–103)	95 (80–106)	0.163
MAP, mmHg	78 (71–86)	73 (68–81)	<0.001	80 (73–89)	75 (69–83)	<0.001
RR, breaths/minutes	19 (17–22)	21 (17–24)	<0.001	20 (18–23)	22 (19–25)	0.003
SpO2,%	97 (96–99)	97 (95–99)	0.010	97 (95–98)	96 (95–98)	0.266
Intervention						
Mechanical ventilation	554 (40.6)	109 (47.2)	0.059	250 (30.5)	78 (54.9)	<0.001
Diuretic use	187 (13.7)	42 (18.2)	0.072	118 (14.4)	35 (24.6)	0.002
RRT	99 (7.2)	52 (22.5)	<0.001	32 (3.9)	22 (15.5)	<0.001
Outcomes						
LOS hospital, day	12.7 (7.3–21.2)	13.5 (6.7–23.5)	0.687	11.9 (6.8–20.5)	8.3 (3.7–17)	<0.001
LOS ICU, day	3.1 (1.9–6.4)	5 (2.5–10.6)	<0.001	3 (1.9–6.2)	4.2 (2.3–8.9)	0.001
BCR category						
High BCR > 26.84	289 (21.2)	85 (36.8)	<0.001	171 (20.8)	49 (34.5)	<0.001

OR, odds ratio; CCI, Charlson Comorbidity Index; SAPS, the simplified acute physiology score; WBC, white blood cells; RBC, red blood cell; MCHC, mean corpuscular hemoglobin concentration; RDW, red blood cell distribution width; BUN, blood urea nitrogen; INR, international normalized ratio; PT, prothrombin time; PTT, partial thromboplastin time; RRT, renal replacement therapy; MAP, mean arterial pressure; RR, respiratory rate; LOS, length of stay; ICU, intensive care unit.

The comparison between survival and deceased cohorts revealed significant differences for patients with PE. Deceased individuals were older with higher CCI and SAPS II scores (*p* < 0.001). Laboratory findings indicated lower hemoglobin, RBC, and platelet levels but higher WBC, glucose, RDW, BUN, creatinine, and BUN/Cr ratio in deceased patients (all *p* < 0.05). Furthermore, deceased patients exhibited lower bicarbonate levels and higher INR and PT values (all *p* < 0.05). Comorbidities such as diabetes, congestive heart failure, coronary artery disease, malignant cancer, renal disease, and cerebrovascular disease were more prevalent among deceased PE patients (all *p* < 0.05). Increased usage of mechanical ventilation, diuretics, and renal replacement therapy was observed in deceased PE patients (all *p* < 0.05). Furthermore, deceased PE patients experienced shorter hospital and ICU stays (all *p* < 0.05). PE patients with a BCR greater than 26.84 had a significantly higher mortality rate compared to those with a lower BCR (*p* < 0.001) (Table 6). In patients with PE, the crude logistic regression analysis indicated that those in the high BCR group had a significantly higher risk of in-hospital mortality compared to those in the low BCR group, with an odds ratio (OR = 2.003, 95% CI: 1.363–2.943, *p* < 0.001). The significant association persisted even after adjusting for potential confounders, with an aOR of 1.585 (95% CI: 1.024–2.45, *p* = 0.039) (Supplementary Table S2). In patients with PE, the Kaplan-Meier survival analysis showed that the median survival time for the high BCR group was 61.306 days (95% CI: 40.380–61.306), significantly shorter than the low BCR group's 97.894 days (95% CI: 85.482–97.894). The high BCR group had an 83% higher risk of in-hospital mortality (HR: 1.8343, 95% CI: 1.2450–2.7028). The log-rank test confirmed a statistically significant difference in survival between the groups (Logrank *p* = 0.0022) (Supplementary Figure S2).

## 4 Discussion

To our knowledge, this study is the first to explore the relationship between BCR and in-hospital mortality among critically ill patients with VTE. Our findings indicate that higher BCR was associated with increased in-hospital mortality among critically ill patients, even after adjusting for potential confounding variables. Furthermore, the baseline BCR levels of the individuals in the deceased cohort were significantly greater than those in the surviving group. Notably, among patients with VTE in critical condition, a BCR greater than 26.84 was found to be an independent risk factor for in-hospital death. Compared to the low BCR group, patients in the high BCR group had significantly shorter median survival times, indicating that BCR may be a prognostic factor for VTE patients, with high BCR indicating a poor prognosis. Moreover, the hazard ratio for the high BCR group was 1.7777, indicating a 77.77% higher risk of in-hospital death in comparison to the low BCR group. Analysis based on VTE types also revealed that patients with high BCR had a significantly increased risk of in-hospital mortality in both the DVT and PE groups. This association remained statistically significant even after adjusting for numerous potential confounders.

Although the kidneys filter both BUN and creatinine, only BUN is reabsorbed in both the proximal and distal renal tubules. The activation of the neurohormonal system, which comprises the sympathetic nervous system, the renin-angiotensin-aldosterone system (RAAS), and vasopressin, impacts the reabsorption of BUN (12). Neurohormonal activation, triggered by conditions like heart failure, VTE, liver cirrhosis, or dehydration, can lead to an elevated BCR even without significant renal dysfunction (30). The assessment of BUN and creatinine levels is a routine practice in the ICU. The BCR is a straightforward and readily obtainable measure, as it relies solely on venous blood samples in clinical settings. Previous studies have reported an association between the BCR and in-hospital mortality in various diseases (17–20). BCR, a biomarker of neurohormonal activity, has been associated with poor prognosis in patients with acute heart failure and is an independent predictor of all-cause mortality (31). Impaired blood flow and endothelial dysfunction in heart failure contribute to a prothrombotic state, increasing the risk of thromboembolic events like stroke, intracardiac thrombi, VTE, PE, and myocardial infarction (32). Chen et al. (33) examined the link between BCR levels and in-hospital mortality among patients with subarachnoid hemorrhage, utilizing the MIMIC-IV database. Their study revealed a significant association, indicating that elevated BCR levels (≥27.208) were correlated with an increased risk of in-hospital mortality in comparison to lower BCR levels (<27.208). Another study by Han et al. (17) examined the correlation between BCR and all-cause mortality in adult patients with septic shock. They demonstrated that even after adjusting for potential confounders, a high BCR (≥27.3 mg/dl) in patients with septic shock was significantly linked to all-cause mortality. Ok et al. (34) investigated the utility of the BUN/Cr ratio upon admission in predicting disease severity and survival rates for patients with COVID-19. Their investigation revealed that a higher BUN/Cr ratio served as an independent prognostic indicator for both the COVID-19 severity and patient outcomes related to survival. Ma et al. (20) conducted a recent investigation to elucidate the association between the BUN/Cr ratio and in-hospital mortality among patients diagnosed with trauma-induced acute respiratory distress syndrome. Their findings revealed a statistically significant correlation between elevated BUN/Cr ratios and an increased risk of death within the hospital setting for these patients. Additionally, the study demonstrated a superior predictive value of the BUN/Cr ratio in predicting in-hospital mortality compared to using BUN or creatinine levels alone. The aforementioned results suggest that a higher BCR is linked to more severe conditions compared to a lower BCR. Our results concur with the above findings. In this cohort study utilizing the MIMIC-IV database, we found that the optimal cut-off value of BCR to predict survival status among patients with VTE was 26.84. Moreover, our findings demonstrated that the BCR serves as an independent predictor of in-hospital death in critically ill VTE patients. Additionally, our results showed a strong association between low BCR levels and improved survival outcomes. Patients in the low BCR group exhibited a statistically significant longer median survival time (86.976 days) than those in the high BCR group (45.477 days). Notably, compared to those with lower BCR levels, a higher BCR level (>26.84) upon admission was correlated with an increased risk of in-hospital mortality in critically ill VTE patients. While additional research is required for a comprehensive understanding of the underlying mechanisms, the elevated BCR in VTE patients could potentially be associated with several mechanisms. Firstly, VTE has been associated with prolonged hypoxemia and hypercapnia, potentially leading to activation of the RAAS (35, 36). While the mechanism is not fully elucidated, this activation may contribute to increased BUN levels through tubular reabsorption of urea.. Secondly, patients with VTE often have concomitant cardiovascular comorbidities, including heart failure (37, 38). In the context of cardiovascular disease, a multifaceted neurohormonal response triggers activation of the renal sympathetic nervous system and the RAAS, potentially leading to altered urea reabsorption (12). Thirdly, VTE is associated with persistent neutrophilia and the subsequent release of proinflammatory mediators due to hypoxia (26). These mediators may disrupt cardiorenal function and neurohumoral regulation, potentially leading to increased BUN levels (30). Finally, VTE can indirectly lead to elevated BUN and creatinine levels by causing blood stasis and reducing blood flow to the kidneys (30).

In the present study, the overall in-hospital mortality of the study population was 14.6%. A study by Ambra et al. (39) in Qatar reported a 13.39% mortality rate for hospitalized patients with venous VTE. Another retrospective cohort study conducted at seven major hospitals in Saudi Arabia demonstrated that the mortality rate for patients with confirmed VTE was 14.3% (40). Using an extensive ICU database, Pisani et al. (41) assessed the risk of VTE in critically ill pneumonia patients and revealed that patients with VTE had a high mortality rate (20.6%). This wide variability in mortality rates among patients with VTE can be attributed to several factors, including the patient population, severity of VTE, comorbidities, and timeliness of diagnosis and treatment. Our findings emphasize the importance of carefully monitoring BCR in VTE patients admitted to ICUs. Since BCR is a simple parameter obtainable from routine clinical blood tests, it may enable clinicians to promptly identify VTE patients prone to adverse outcomes, necessitating early intensive care intervention. There are several notable strengths in this study. To our knowledge, it is the first to investigate the potential association between BCR and in-hospital mortality among VTE patients using a large dataset from a heterogeneous population in the ICU. Furthermore, even after adjusting for various confounding factors in three different models, the relationship between the BCR and mortality remained consistent, suggesting the robustness and stability of our results. The BCR is a readily available index for risk assessment in critically ill patients, as BUN and creatinine are part of routine blood tests. Furthermore, this research suggests clinicians can enhance prognostic accuracy for patients with VTE by utilizing this affordable and easily accessible biomarker.

This study has several limitations. Firstly, although multivariable analyses were employed, the findings of this study could still be affected by residual bias and unmeasured confounding factors due to its retrospective nature. Secondly, retrospective studies have inherent limitations, such as potential missing data and an inability to establish causality. These factors may impact the validity and interpretation of our findings. Thirdly, owing to constraints within the MIMIC-IV database, information regarding several factors affecting the BCR, such as the administration of corticosteroids and specific antibiotics, protein consumption, and muscle mass, were not evaluated in this study because they could not be extracted from the MIMIC-IV database. Fourthly, the ROC curve analysis was used to identify the optimal threshold value for the BCR, with the maximal Youden index serving as the predictor of survival status. Nevertheless, the area under the curve for BCR was smaller than expected. Finally, the MIMIC-IV database lacks detailed information regarding mortality causes and specific treatments such as anticoagulation and thrombolysis, which restricts our ability to assess potential differences in therapeutic approaches among VTE patients. Therefore, prospective cohort studies are warranted to validate these findings. Finally, this was a single-center study based on data obtained from the MIMIC-IV database, limiting our findings' generalizability.

## 5 Conclusions

Our findings demonstrate that the BCR serves as an independent predictor of mortality within the hospital setting for critically ill patients diagnosed with VTE. Patients presenting with elevated BCR levels exhibited a significantly increased risk of in-hospital mortality compared to those with lower BCR levels. BCR is a highly practical, readily available, and relatively inexpensive index that could be a valuable tool for clinicians in managing and stratifying the risk for critically ill VTE patients at an early stage.

## Data Availability

Publicly available datasets were analyzed in this study. This data can be found here: The Medical Information Mart for Intensive Care IV (MIMIC-IV) database at https://physionet.org/content/mimiciv/2.2/.
