# Supplementary material for: Blood urea nitrogen to creatinine ratio is associated with in-hospital mortality in critically ill patients with venous thromboembolism: a retrospective cohort study
Source: Front Cardiovasc Med. 2024 Jun 13;11:1400915. doi: 10.3389/fcvm.2024.1400915 (PMC11208632; doi:10.3389/fcvm.2024.1400915)
Supplement: Supplementary file 1 [file Datasheet1.pdf]

Supplementary Table 1: International Classification of Diseases (ICD) codes used to identify Venous Thromboembolism

ICD-9-CM codes	ICD-10-CM codes
45119, 4512, 45181, 45182, 45183, 45184, 45189, 4519, 4532, 4538, 45381, 45382, 45383, 45384, 45385, 45386, 45387, 45389, 4539, 4150, 41511, 41512, 41513, 41519, 45340, 45341, 45342, 4510, 452, 4530, 4531, 4533	I808, I809, I8290, I82890, I2699, I2692, I2690, I2602, I2609, I8000, I8001, I8002, I81, I820, I821

Supplementary Table 2. Logistic regression analyses of the BCR groups for in-hospital mortality in patients with deep vein thrombosis and Pulmonary embolism.

	**Deep Vein thrombosis**				**Pulmonary embolism**			
**Characteristic**	**Crude Model**		**Adjusted model**		**Crude model**		**Adjusted model**	
	OR (95% CI)	p-Value	aOR (95% CI)	p-Value	OR (95% CI)	p-Value	aOR (95% CI)	p-Value
Low BCR group	1 (Reference)		1 (Reference)		1 (Reference)		1 (Reference)	
High BCR group	2.170 (1.612–2.921)	<0.001	1.872 (1.330–2.634)	<0.001	2.003 (1.363–2.943)	<0.001	1.585 (1.024–2.453)	0.039

Crude model: No adjustment. Adjusted model: Adjusted for age, hemoglobin, white blood cells, glucose, Mean corpuscular hemoglobin concentration, Red cell distribution width, hematocrit, bicarbonate, International normalized ratio, diabetes, congestive heart failure, coronary artery disease, renal disease, malignant cancer, cerebrovascular disease, mechanical ventilation, diuretic use, and renal replacement therapy. BCR; Blood urea nitrogen to creatinine ratio.

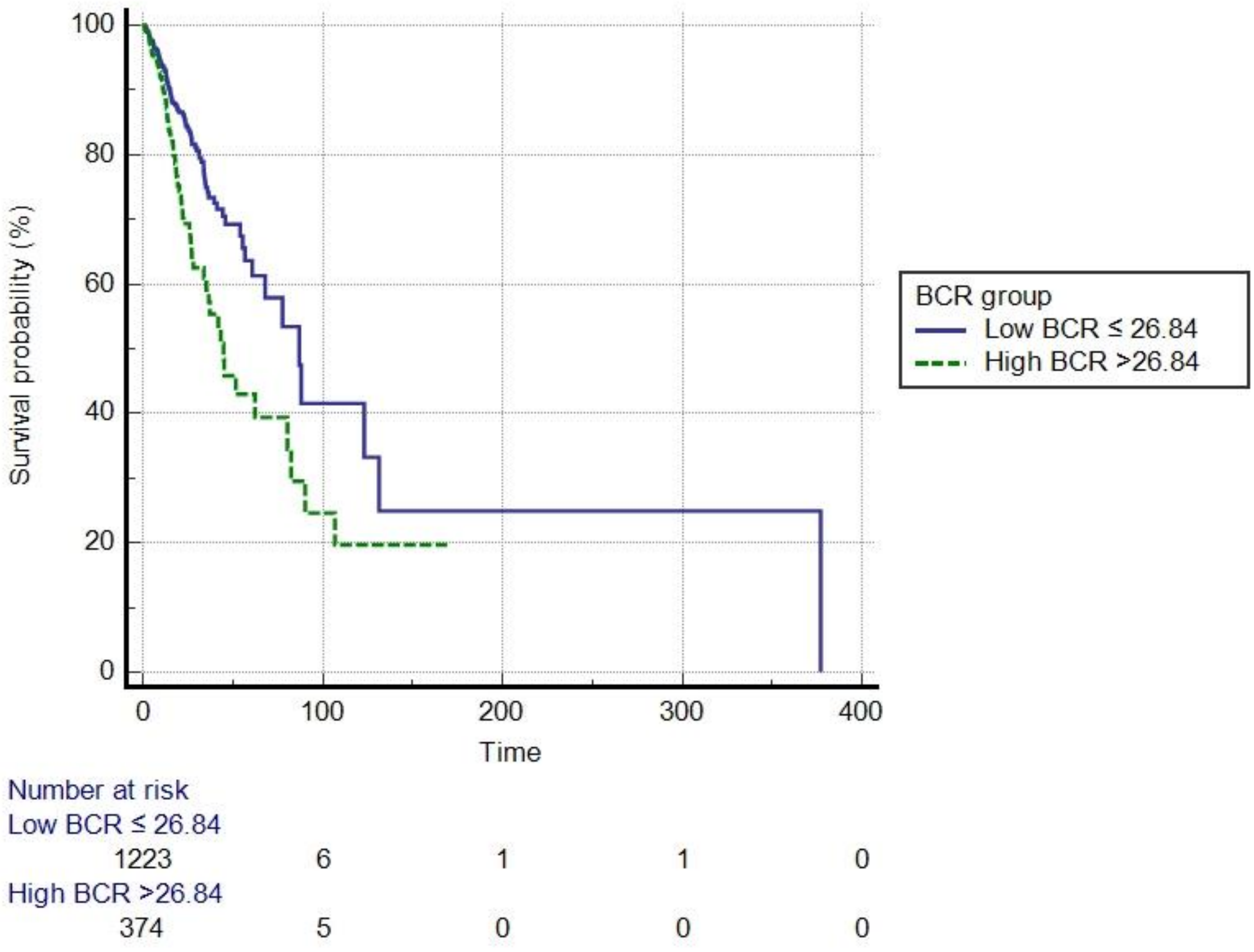

Supplementary Figure 1. Kaplan-Meier survival curve for in-hospital mortality for the high and low BCR groups in patients with deep vein thrombosis. BCR, Blood urea nitrogen to creatinine ratio.

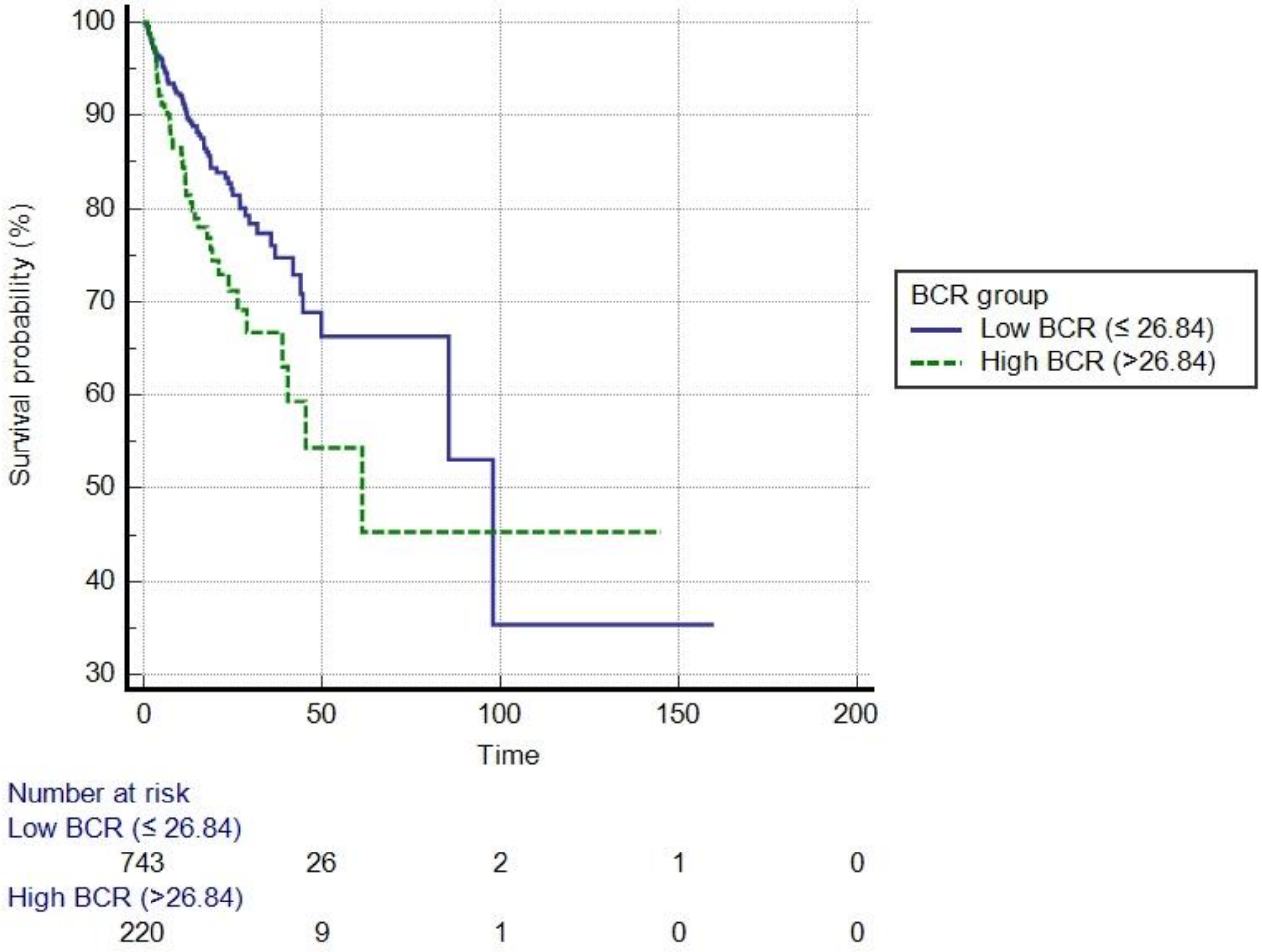

Supplementary Figure 2. Kaplan-Meier survival curve for in-hospital mortality for the high and low BCR groups in patients with pulmonary embolism. BCR, Blood urea nitrogen to creatinine ratio.
